# Supplementary material for: FERN – a Java framework for stochastic simulation and evaluation of reaction networks
Source: BMC Bioinformatics. 2008 Aug 29;9:356. doi: 10.1186/1471-2105-9-356 (PMC2553347; doi:10.1186/1471-2105-9-356)
Supplement: Additional file 1 — FERN distribution, Version 1.3. This archive contains the FERN source code and binaries as well as documentation and example models in FernML and SBML. [file 1471-2105-9-356-S1.zip › fern/doc/javadoc/fern/tools/ConfigReader.html]

ConfigReader


---


|  |  |  |
| --- | --- | --- |
| **Overview** \| **Package** \| **Class** \| **Use** \| **Tree** \| **Deprecated** \| **Index** \| **Help** | |  |
| **PREV CLASS**   **NEXT CLASS** | **FRAMES**    **NO FRAMES**     **All Classes** |  |
| SUMMARY: NESTED \| FIELD \| CONSTR \| METHOD | DETAIL: FIELD \| CONSTR \| METHOD |  |


---


## fern.tools Class ConfigReader

```
java.lang.Object
  fern.tools.ConfigReader
```

---

``` public class ConfigReader extends Object ```

Reads simple config files for test cases and makes the data of it accessible.

The file has to contain lines like  
`field=value`

If a line starts with // or # it is ignored.

**Author:**
:   Florian Erhard

---

**Constructor Summary**

`ConfigReader(String fn)`
  
          Reads the given file and stores the data.


| **Method Summary** | |
| --- | --- |
| `double` | `getAsDouble(String key)`             Gets the value of the line `key=value` as double |
| `int` | `getAsInt(String key)`             Gets the value of the line `key=value` as int |
| `String` | `getAsString(String key)`             Gets the value of the line `key=value` as string |
| `String[]` | `getAsStringArr(String key)`             Gets the value of the line `key=value` as string array (the value is simply splitted by ,) |

**Methods inherited from class java.lang.Object**

`clone, equals, finalize, getClass, hashCode, notify, notifyAll, toString, wait, wait, wait`

**Constructor Detail**

### ConfigReader

```
public ConfigReader(String fn)
             throws IOException
```

:   Reads the given file and stores the data.

    **Parameters:**: `fn` - config file **Throws:**: `IOException`


**Method Detail**

### getAsString

```
public String getAsString(String key)
```

:   Gets the value of the line `key=value` as string

    :   **Parameters:**: `key` - name of the field **Returns:**: value

---


### getAsStringArr

```
public String[] getAsStringArr(String key)
```

:   Gets the value of the line `key=value` as string array (the value is simply splitted by ,)

    :   **Parameters:**: `key` - name of the field **Returns:**: value

---


### getAsDouble

```
public double getAsDouble(String key)
```

:   Gets the value of the line `key=value` as double

    :   **Parameters:**: `key` - name of the field **Returns:**: value

---


### getAsInt

```
public int getAsInt(String key)
```

:   Gets the value of the line `key=value` as int

    :   **Parameters:**: `key` - name of the field **Returns:**: value


---


|  |  |  |
| --- | --- | --- |
| **Overview** \| **Package** \| **Class** \| **Use** \| **Tree** \| **Deprecated** \| **Index** \| **Help** | |  |
| **PREV CLASS**   **NEXT CLASS** | **FRAMES**    **NO FRAMES**     **All Classes** |  |
| SUMMARY: NESTED \| FIELD \| CONSTR \| METHOD | DETAIL: FIELD \| CONSTR \| METHOD |  |


---
